# Relevance of inflammatory pseudotumor associated with bladder tumor

**Published:** 2007

**Authors:** Annamma Kurien, Joseph Thomas, Sreedhar Reddy, Arun Chawla

**Affiliations:** Department of Pathology, Melaka Manipal Medical College, Manipal, India; * Department of Urology, Kasturba Medical College, Manipal, India. E-mail: drjosephthomas@yahoo.co.in

## SUMMARY

Inflammatory pseudotumors (IPT) are sometimes seen in the follow-up bladder biopsies of patients with transitional cell carcinoma. The relevance of this finding was studied in 809 patients who were on surveillance after the initial definitive treatment for superficial transitional cell carcinoma of the bladder. During this period, there were 16 (2%) patients who had bladder mass but without evidence of malignancy. They had histological evidence of only IPT—spindle cells and inflammatory infiltrate. In all these patients, the primary tumor was a high-grade transitional cell carcinoma.Fifteen of these patients had received adjuant intravesical therapy during the initial treatment. This included intravesical immunotherapy with bacillus Calmette-Guerin (BCG) in 13, intravesical chemotherapy in one and chemoradiation in one. The median follow-up period was 26 months after the diagnosis of IPT. Twelve patients (75%) showed tumor recurrence within a median period of 16 months. All the recurrences were high-grade urothelial carcinoma. Nine (56%) progressed to a higher stage with a median time to progression of seven months from the diagnosis of IPT. Four patients (25%) had evidence of metastases upon diagnosis of recurrence and two more patients developed metastatic disease later. Eventually 12 patients died within a median period of 26 months after the diagnosis of IPT.

## COMMENTS

It is common to see desmoplastic stromal reactions in patients with carcinomas of the breast, ovary, prostate, pancreas, lungs and the gastrointestinal tract (GIT).[1] Inflammatory pseudotumors (IPT) of the bladder are thought to be a benign condition, often associated with infections and repeated bladder surgeries. The finding of a bladder mass histologically compatible with IPT could be a desmoplastic-like stromal reaction to an underlying concomitant malignancy.[2] The relevance of this finding during the follow-up period is not well known. The current study finds that the rate of tumor progression and period for progression was shorter compared with those in literature with BCG instillations.[3] The short time to progression suggests that advanced disease could have been present at the time of the IPT diagnosis. The growth factors produced by the tumor cells could have activated dormant fibroblasts into myofibroblasts. These tumors have an aggressive biological nature, both locally and systematically.[4] Hence, to monitor the development of underlying aggressive urothelial malignancy, it is important to take frequent bladder biopsies and multiple sections from different tissue levels in patients with IPT during the follow-up period. It is possible that an early diagnosis and adequate treatment could have improved the dismal prognosis noted in the study. The absence of a comparative control group is the major drawback in this study. There is a high possibility that the finding of IPT could also have been a coincidence.
